# 

**Published:** 1872-12

**Authors:** 


### ATLANTA MEDICAL & SURGICAL JOURNAL.

EDITED BY

JOSEPH P. LOGAN, M.D.,

AND

W. F. WESTMORELAND, M.D.,

*Professor of the Principles and Practice of Surgery in Atlanta Medical College.*

VOL. X.—DECEMBER, 1872.—No. 9.

*PAX ET SCIENTIA, SED VERITAS SINE TIMORE.*

ATLANTA, GEORGIA:

HERALD PUBLISHING COMPANY'S PRESS.

1872.

